# Transcriptomics analysis of LINC02202/XBP1 axis in melanoma: Implications for drug targeting and PD‐1 monoclonal antibody efficacy

**DOI:** 10.1111/jcmm.18247

**Published:** 2024-03-23

**Authors:** Yuanyuan Shang, Haiqian Yang, Jian Cui, Lipeng Wang, Le Wang, Yuan Wang, Miao‐Miao Zhao, Pei‐Yao Yu, Hui Qiao, Wen‐Jun Yang

**Affiliations:** ^1^ School of Public Health Ningxia Medical University Yinchuan China; ^2^ Ningxia Medical University Yinchuan China; ^3^ Department of Anesthesia General Hospital of NingXia Medical University Yinchuan China; ^4^ Department of Dermatology General Hospital of Ningxia Medical University Yinchuan China; ^5^ Pathology Department The First Affiliated Hospital, Hainan Medical University Haikou China; ^6^ Cancer Institute The General Hospital of Ningxia Medical University Yinchuan China

**Keywords:** immunotherapy, LINC02202/XBP1axis, melanoma, PD‐1

## Abstract

Malignant melanoma (MM) is a highly aggressive and deadly form of skin cancer, primarily caused by recurrence and metastasis. Therefore, it is crucial to investigate the regulatory mechanisms underlying melanoma recurrence and metastasis. Our study has identified a potential targeted regulatory relationship between LINC02202, miR‐526b‐3p and XBP1 in malignant melanoma. Through the regulation of the miR‐526b‐3p/XBP1 signalling pathway, LINC02202 may play a role in tumour progression and immune infiltration and inhibiting the expression of LINC02202 can increase the efficacy of immunotherapy for melanoma. Our findings shed light on the impact of LINC02202/XBP1 on the phenotype and function of malignant melanoma cells. Furthermore, this study provides a theoretical foundation for the development of novel immunotherapy strategies for malignant melanoma.

## 1 INTRODUCTION

Malignant melanoma (MM) is a highly aggressive and lethal form of skin cancer, with its incidence increasing at a rate of approximately 3%–5% annually.^1^, ^2^ Although melanoma accounts for less than 5% of skin malignant tumours, it is responsible for 80% of skin malignant tumour deaths, posing a significant threat to human health.^3^ Surgical resection is the preferred treatment for early localized lesions, but the risk of postoperative metastasis remains high. For patients with advanced metastatic malignant melanoma, systemic treatment is necessary as traditional radiotherapy and chemotherapy are not effective due to the cells' resistance, leading to a high mortality rate.^4^

Recurrence and metastasis are the primary causes of death in malignant melanoma. During early metastasis, tumour cells acquire the ability to migrate, invade and spread to distant tissues and organs by crossing the surrounding stroma and entering the circulation.^5^ Approximately 25%–33% of melanomas arise directly from benign melanocytic nevi, which are tumours caused by the proliferation of melanocytic cells with oncogenic mutations. Melanomas occurring on exposed skin, such as the head and neck, are rarely associated with moles, indicating a fundamental genetic difference from melanomas occurring on nonexposed skin.^6^ The role of LINC02202 in malignant melanoma remains unknown, although it has been identified as a risk factor affecting the prognosis of hepatocellular carcinoma and potentially associated with adipogenic differentiation of ASCs.^7^, ^8^

Immunotherapy has shown promising results in the treatment of advanced and metastatic cancers, leading to its approval as adjuvant or adjunctive therapy for non‐small cell lung cancer, melanoma, triple‐negative breast cancer and other solid tumours.^9^ Therefore, investigating the regulatory mechanisms underlying immunotherapy efficacy is of significant clinical importance.

In our study, we sequenced the long noncoding RNAs of malignant melanoma developed from melanocytic nevus at various sites and performed differential gene expression analysis. LINC02202 was identified as one of the top differentially expressed genes, and it was found to regulate the expression of PIK3R1 and FOXO1 in the PI3K signalling pathway. We also discovered a potential targeted regulatory relationship between LINC02202, miR‐526b‐3p and XBP1 in malignant melanoma. LINC02202 may further regulate immune infiltration and the efficacy of immune checkpoint treatments by modulating the miR‐526b‐3p/XBP1 signalling pathway, contributing to immune evasion, proliferation and metastasis of malignant melanoma. Our study provides insights into the effects of LINC02202/XBP1 on the phenotype and function of malignant melanoma cells, offering a theoretical basis for the development of new immunotherapy strategies for this aggressive cancer.

## 2 MATERIALS AND METHODS

### 2.1 Cell lines

The human foreskin fibroblasts cell line (HFF), the three human melanoma cell lines (A375, M21 and SKMEL‐5) and one mouse melanoma cell lines B16 used in this study were obtained from the Chinese typical culture preservation center. These cell lines were cultured in RPMI 1640 medium (Gibco) supplemented with 10% foetal bovine serum. The cells were maintained at a temperature of 37°C in a humidified atmosphere with 5% CO_2_.

### 2.2 miRNA, SiRNA and plasmid transfection

The sequences of the siRNAs and mRNA used in this study are listed in Table 1. SiRNAs targeting LINC02202 and miRNA mimics and inhibitors were designed and synthesized by Ruibo Guangzhou. The transfection methods for introducing these siRNAs and miRNA mimics/inhibitors into cells were performed according to the references.^10^ Please refer to the cited reference for detailed information on the transfection methods used in this study.

**TABLE 1:** The primer sequences information.

Genes		Sequence
miR‐526b‐3p	mimic	5′‐GAAAGUGCUUCCUUUUAGAGGC‐3′
miR‐526b‐3p	mimic NC	5′‐CGAUCGCAUCAGCAUCGAUUGC‐3′
miR‐526b‐3p	inhibitor	5′‐GCCUCUAAAAGGAAGCACUUUC‐3′
miR‐526b‐3p	inhibitor NC	5′‐UGAGCUGCAUAGAGUAGUGAUUA‐3′
miR‐526b‐3p	Forward	5′‐GGGGAGTTAGGATTAGGTC‐3′
	Reverse	5′‐TGCGTGTCGTGGAGTC‐3′
U6	Forward	5′‐GCTCGCTTCGGCAGCACAT‐3′
	Reverse	5′‐AAAATATGGAACGCTTCACG‐3′
GAPDH	Forward	5′‐CGCTCTCTGCTCCTCCTGTTC‐3′
	Reverse	5′‐ATCCGTTGACTCCGACCTTCAC‐3′
LINC02202	Forward	5′‐AGCTTGGCTACTGGGTCCAT‐3′
	Reverse	5′‐TGACAGGAGACAGGCTGTGA‐3′
XBP1	Forward	5′‐CCTGGTTGCTGAAGAGGAGG‐3′
	Reverse	5′‐CCATGGGGAGATGTTCTGGAG‐3′
siLINC02202	Sense	5′‐GCATCCTGGCCATTGCAGTCCTTTA‐3′
	Antisense	5′‐GCAGTCCCGTTAACGCTGTCTCTTA‐3′

### 2.3 The immunohistochemistry (IHC) and immunofluorescence (IF) analysis

IHC and IF staining was performed with indicated antibodies using the standard protocol. The human anti‐XBP1 antibody (1:500 dilution, 24,168‐1‐AP, Proteintech) and anti‐PD‐L1 antibody (1:100 dilution, ab205921, Abcam). Ki67 (1:100 dilution, ab15580, Abcam), respectively, as indicated. The IHC‐stained tissue sections were performed as described previously.^11^ After IF analysis, the colocalization between proteins were measured using Image J 1.53 t (National Institutes of Health, USA).

### 2.4 Western blotting

After transfection, the cells were collected and lysed for further analysis. Western blot analysis was performed following the methods described in the provided references.^12^ The primary antibodies used in this study were diluted as follows: Cleaved‐caspase‐3 (1:1000, ab32042, Abcam), XBP1 (1:1500, 24,168‐1‐AP, Proteintech) and GAPDH (1:5000, ab9485, Abcam). Please refer to the cited references for more detailed information on the Western blot analysis methods used in this study.

### 2.5 Transcriptome sequencing

Collect human tissue samples from five pairs of melanomas and three pairs of pigmented nevi, fix them with formalin, and send them to Shanghai Yuanshen Gene Company for transcriptome sequencing. Analyse based on R language pack.

### 2.6 RNA extraction and quantitative real‐time PCR

RNA extraction from cells or tissues was performed using TRIzol reagent (Invitrogen, Carlsbad, CA) following the manufacturer's instructions. The extracted RNA was then subjected to quantitative reverse transcription polymerase chain reaction (qRT‐PCR) analysis. The expression levels of LINC02202, has‐miR‐526b‐3p, and RNU6 were measured using qRT‐PCR with primer sets obtained from RiboBio (Guangzhou, China). The qRT‐PCR method used in this study can be found in the provided reference.^13^ The primer sequences for qRT‐PCR are listed in Table 1.

### 2.7 CCK‐8 proliferation assay

After transfection, cell proliferation was evaluated using the Cell Counting Kit‐8 (CCK‐8) assay from Promega. The CCK‐8 assay measures cell viability and proliferation based on the ability of viable cells to convert a tetrazolium salt (WST‐8) into a coloured formazan product. The assay was performed according to the manufacturer's instructions provided by Promega. The absorbance of the formazan product was measured using a microplate reader at the appropriate wavelength. The CCK‐8 assay provides a quantitative measurement of cell proliferation and viability, allowing for the assessment of the effects of transfection on cell growth.

### 2.8 Flow cytometric analyse apoptosis

Apoptosis assay was performed using the FITC Annexin V Apoptosis Detection Kit I from BD Biosciences, following the manufacturer's protocol. The assay detects apoptotic cells by labelling them with FITC‐conjugated Annexin V, which binds to phosphatidylserine exposed on the outer membrane of apoptotic cells. Stained cells were then analysed using a FACSCalibur Flow Cytometer from BD Biosciences. This flow cytometer allows for the quantification and characterization of apoptotic cells based on their fluorescence properties. Please refer to the provided reference^12^ for more detailed information on the apoptosis assay and flow cytometry analysis used in this study.

### 2.9 Transwell assay

After processing, the cells were stained with a 0.4% crystal violet solution. This staining method allows for the visualization and quantification of cells. The invading cells were imaged using a digital microscopy system, specifically a Nikon microscope. The assay utilized 8.0 μm Transwell Permeable Supports from Corning. These Transwell inserts have a porous membrane with a pore size of 8.0 μm, allowing for the study of cell invasion through the membrane. The stained cells were observed and imaged using the digital microscopy system, enabling the analysis of cell invasion.

### 2.10 Luciferase reporter assay

The experiments were performed in triplicate to ensure the reliability of the results. Luciferase activity was measured using the dual‐luciferase assay system from Promega (Madison, WI). In this assay, 293 T cells were co‐transfected with different combinations of reporter plasmids, including pmirGLO‐LINC02202‐WT, pmirGLO‐LINC02202‐MUT, pmirGLO‐miR‐has‐miR‐526b‐3p‐UTRWT or pmirGLO‐has‐miR‐526b‐3p‐3'UTR‐MUT, along with the corresponding mimics, such as mimics NC or has‐miR‐526b‐3p mimics. After 48 h of incubation, the cells were subjected to a luciferase reporter assay. This assay allows for the quantification of luciferase activity as an indicator of gene regulation. Please refer to the provided reference^13^ for more detailed information on the luciferase reporter assay used in this study.

### 2.11 Transplanted tumour model

In the transplanted tumour model, B16 cells that were stably transfected with lentiviruses were constructed in the laboratory. These transfected B16 cells were cultured and prepared for injection. C57 mice were divided into four groups, with five mice in each group. Each mouse was injected with 5 × 10^6^ cells in a volume of 100 μL. Additionally, the mice received anti‐PD1 antibody treatment at a dose of 200 μg per mouse. The experiment was conducted until the tumour volume reached 1500 mm^3^, at which point the mice were sacrificed 15 days after injection.^12^ All animal experiments were performed in accordance with the ethical policies and procedures approved by the Laboratory Animal Welfare and Ethics Committee of the Ningxia medical university.

### 2.12 Statistical analysis

For statistical analyses, unpaired Student's *t*‐tests or one‐way ANOVA followed by Duncan's multiple range tests were performed using Prism 8.0 software. A *p*‐value less than 0.05 was considered statistically significant. Kaplan–Meier survival analysis was used to calculate the overall survival rate of melanoma. This analysis helps assess the impact of different treatments on the survival of mice in the study.

## 3 RESULTS

### 3.1 LINC02202 is highly expressed in melanoma patients' tissues

**FIGURE 1:**
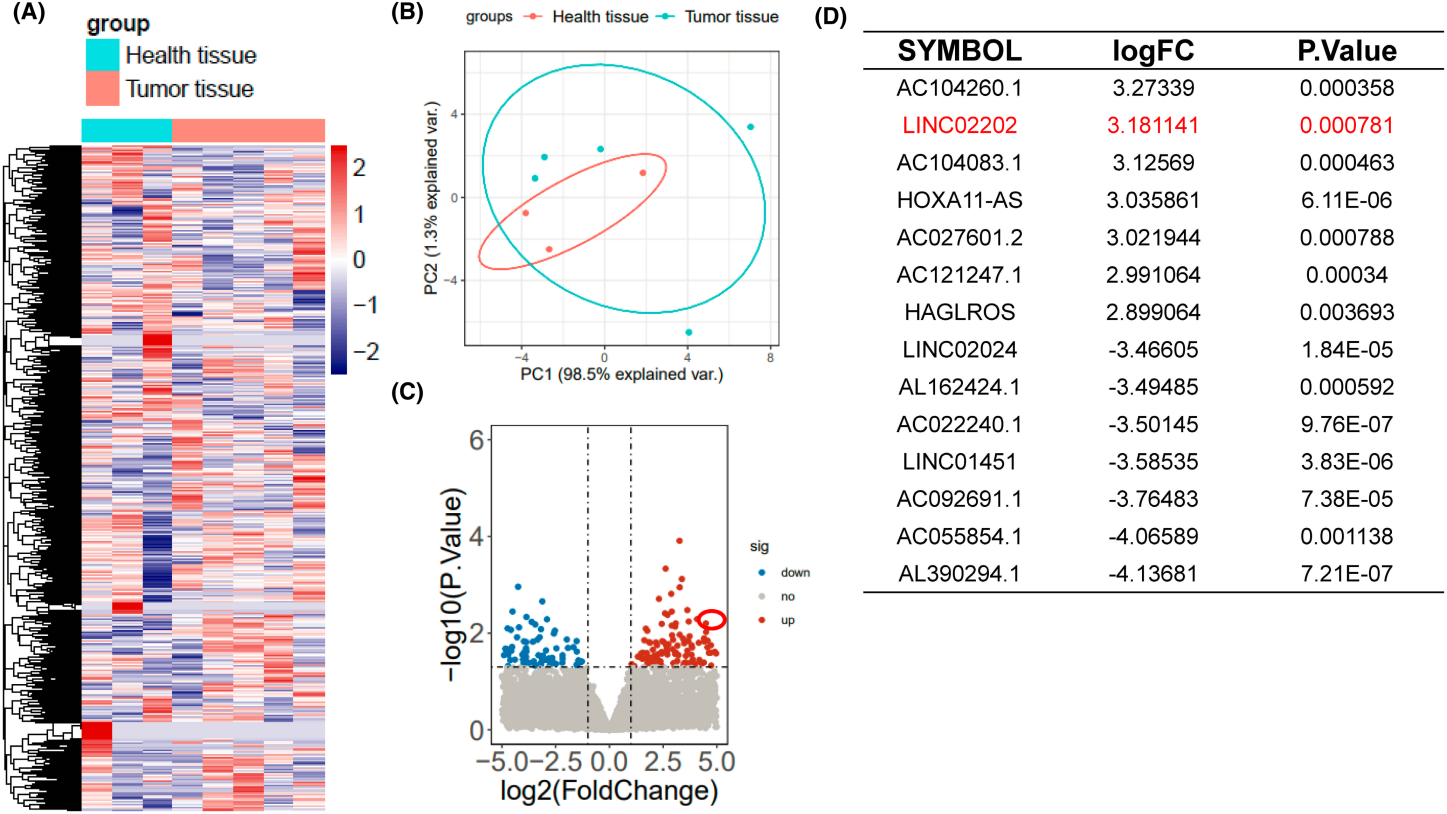
LINC02202 is increased in melanoma patients. (A) Heat map showing melanocytic nevi tissue and five melanoma patients' tissues groups all noncoding RNA expression. (B) PCA analysis showed the difference between the melanoma and melanocytic nevi. (C) Volcano plot analysis and lncRNA differential analysis of the differentially expressed noncoding RNAs. (D) The top seven list of increased and decreased expression difference analysis.

Based on the sequencing results from three melanocytic nevi tissues and five melanoma patients' tissues, bioinformatics analysis was performed. A heat map was generated to visualize the differential expression of all noncoding RNAs between the two groups (Figure 1A). PCA analysis was conducted to demonstrate the distinction between melanoma and melanocytic nevi (Figure 1B). Volcano plot analysis and lncRNA differential analysis identified LINC02202 as a highly expressed lncRNA in melanoma patients compared to the melanocytic nevi group (Figure 1C). LINC02202 ranked among the top 10 highly expressed lncRNAs, with a statistically significant *p*‐value of 0.000781 (Figure 1D).

### 3.2 LINC02202 suppress melanoma cell growth and metastasis

**FIGURE 2:**
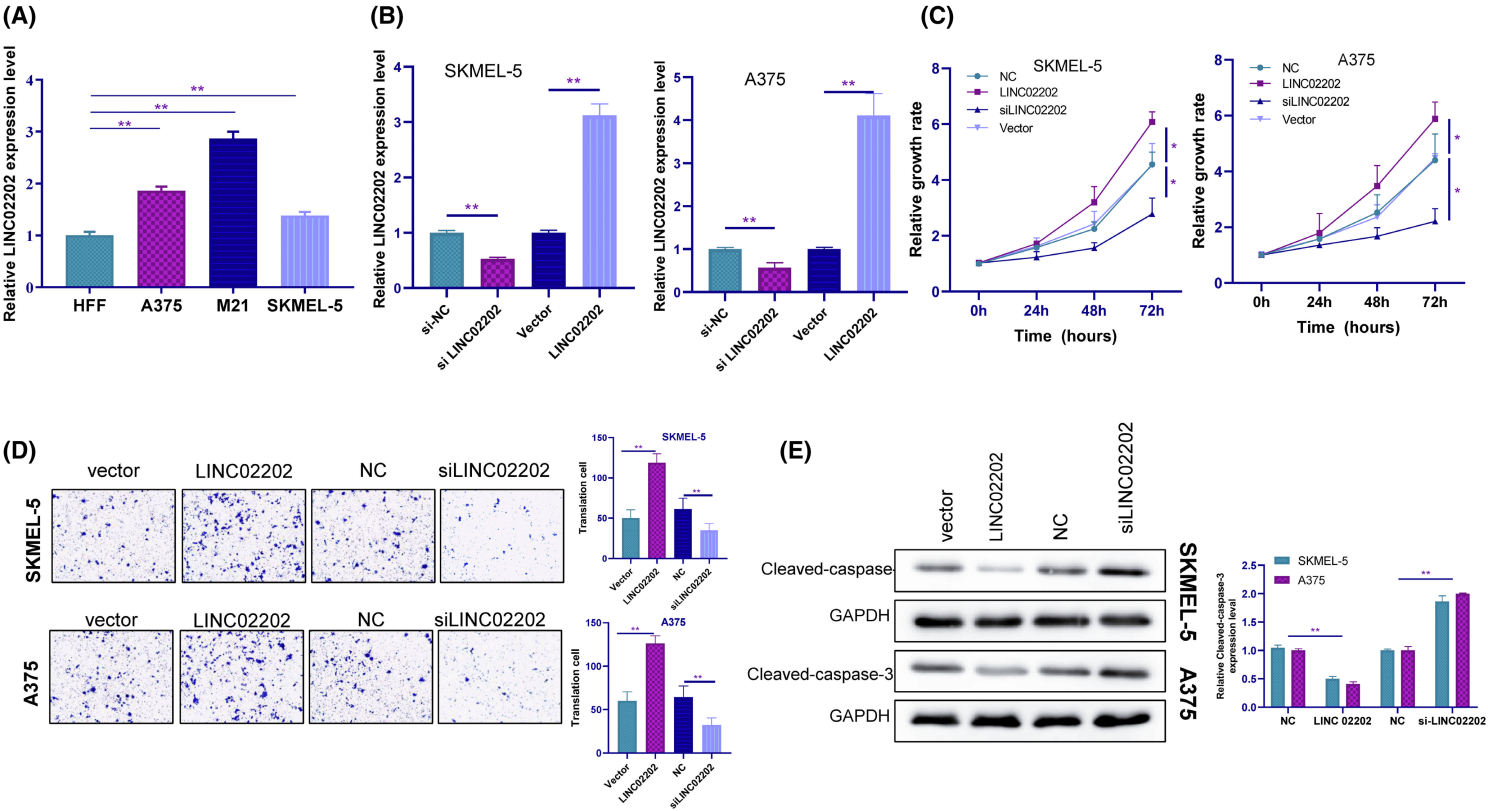
LINC02202 suppress melanoma cell viability, apoptosis and metastasis. (A) The RT‐PCR detected the relative expression of LINC02202 in the three melanoma cell lines (A375, M21 and SKMEL‐5) and the normal cell line human foreskin fibroblasts (HFF). (B) We transfected siRNA and LINC02202 in A375 and SKMEL‐5 cells and using RT‐PCR detected LINC02202 expression at 72 h post transfection. (C) CCK‐8 assays analysed the effect of LINC02202 on cell viability. (D) Transwell assays analysed the effect of LINC02202 on cell migration. (E) The Western blotting assay analysed the effect of LINC02202 on cell apoptosis. *p < 0.05, **p<0.01, ***p < 0.001.

The relative expression of LINC02202 was found to be significantly higher in the three melanoma cell lines (A375, M21 and SKMEL‐5) compared to the normal cell line human foreskin fibroblasts (HFF) (Figure 2A). To investigate the function of LINC02202 in melanoma tumourigenesis, the A375 and SKMEL‐5 cell lines, which exhibited intermediate expression of LINC02202, was used. SiRNA and LINC02202 were transfected into A375 and SKMEL‐5 cells, and LINC02202 expression was detected 72 h post transfection (Figure 2B). CCK‐8 assays revealed that cell viability was significantly reduced in LINC02202‐overexpressing cells (Figure 2C). Migration assays showed a higher migration rate in LINC02202‐overexpressing cells, while a lower migration rate was observed when LINC02202 was knocked down (Figure 2D). Western blotting further supported these findings, as the expression of Cleaved‐caspase‐3 was induced in the siLINC02202 group and suppressed in the LINC02202‐overexpressing group (Figure 2E).

### 3.3 MiR‐526b‐3p is an important target of LINC02202

**FIGURE 3:**
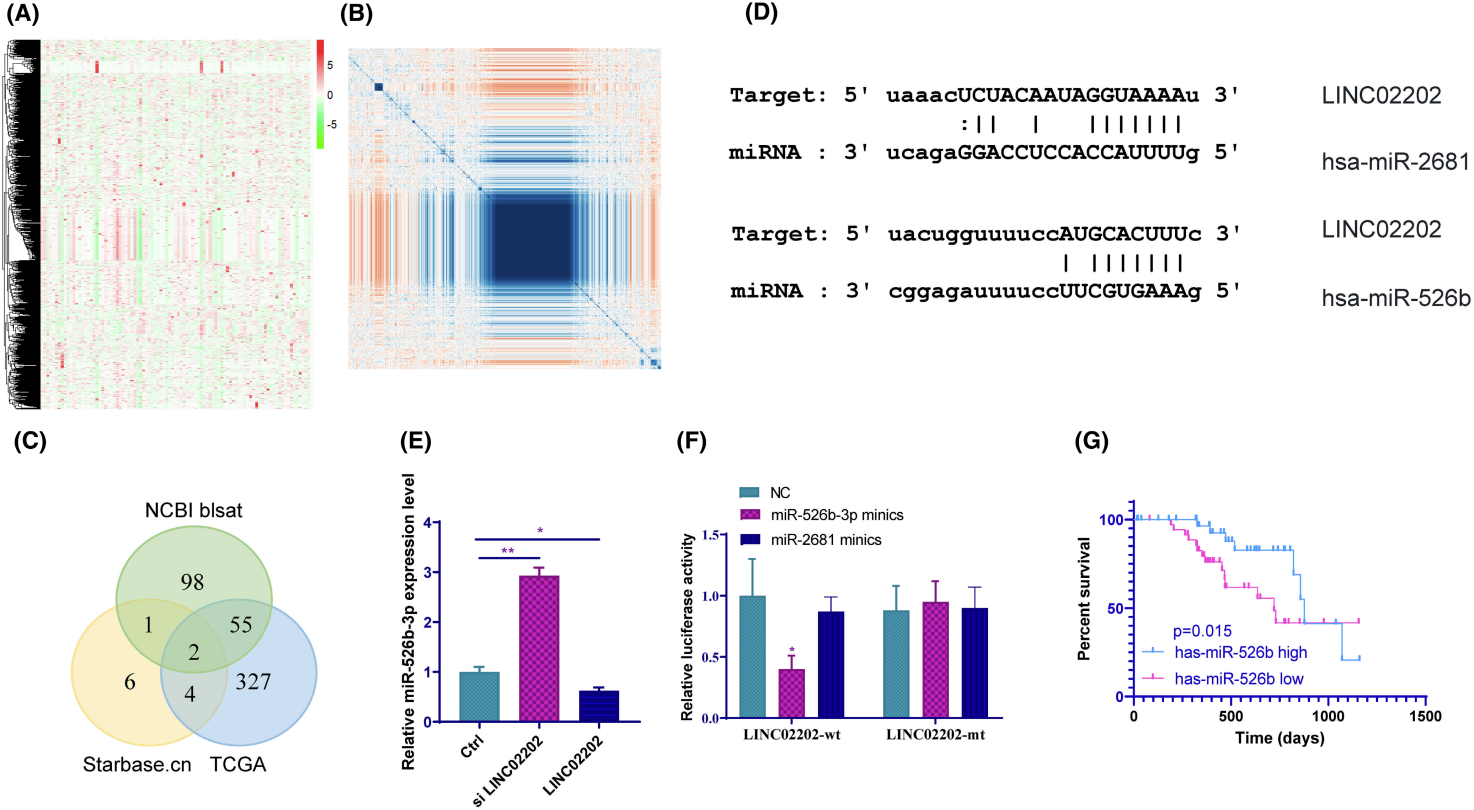
LINC02202 can target MiR‐526b‐3p. (A) The miRNA expression of each sample in the TCGA database were shown by hot map. (B) The correlation analysis between each miRNA and LINC02202. (C) The intersection of correlation analysis, NCBI Blast results and TCGA database prediction analysis results. (D) Predicted binding site for 3'UTR of hsa‐miR‐2681 and has‐miR‐526b‐3p and LINC02202 sequences. (E) The RT‐PCR detected the miR‐526b‐3p expression level 72 h post transfection. (F) Luciferase reporter assay for LINC02202 that directly targets miR‐526b‐3p. (G) The impact of miR‐526b‐3p on the survival and prognosis of melanoma by TCGA database. *p < 0.05, **p<0.01.

In our study, we utilized the TCGA database to search for miRNA expression in melanoma samples. A heatmap was generated to visualize the miRNA expression in each sample (Figure 3A). Correlation analysis was performed between each miRNA and LINC02202, resulting in the identification of two miRNAs: hsa‐miR‐2681 and has‐miR‐526b‐3p (Figure 3B–D). While hsa‐miR‐2681 has limited research, it is almost nonexpressed in the TCGA database, so we focused on has‐miR‐526b‐3p, which has been reported in glioma and colorectal cancer but rarely in melanoma. We transfected LINC02202 or siLINC02202 into SKMEL‐5 cells and detected the expression level of miR‐526b‐3p 72 h post transfection, finding that miR‐526b‐3p expression increased when LINC02202 was disturbed (Figure 3E). A luciferase reporter assay demonstrated significantly lower luciferase activity in the miR‐526b‐3p mimic+ LINC02202‐wt group compared to the miR‐526b‐3p mimic+ LINC02202‐mut group (Figure 3F). Additionally, there was greater LINC02202 enrichment in the bio‐miR‐526b‐3p group, indicating a strong correlation (Figure 3F). Furthermore, survival analysis based on TCGA data showed a positive correlation between miR‐526b‐3p expression and good prognosis (Figure 3G).

### 3.4 MiR‐526b‐3p suppressed melanoma progression

**FIGURE 4:**
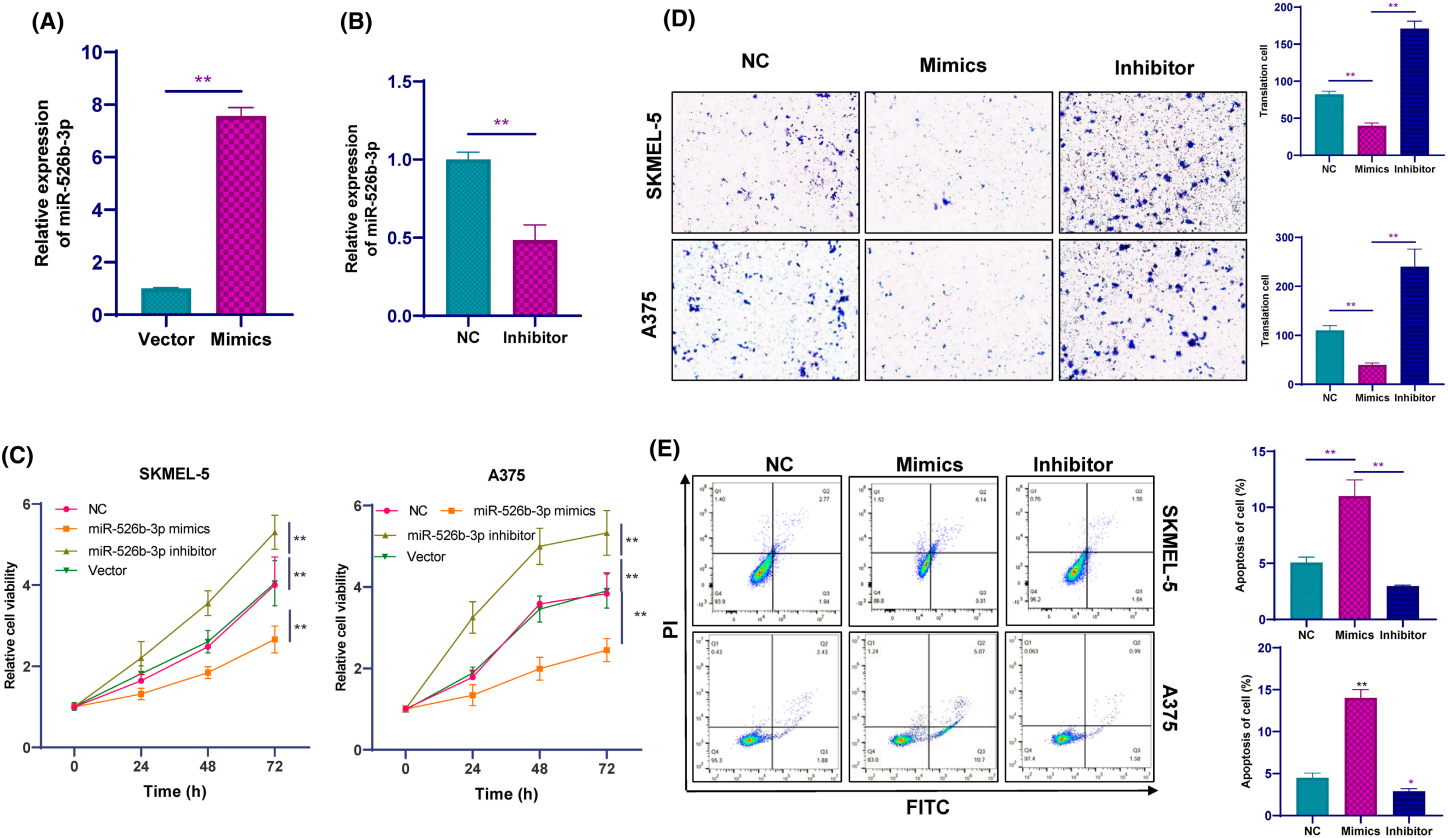
MiR‐526b‐3p could regulate melanoma progression. (A, B) The RT‐PCR detected miR‐526b‐3p expression levels 72 h post transfection. (C) CCK‐8 assays analysed the effect of miR‐526b‐3p on cell viability. (D) Transwell assays analysed the effect of miR‐526b‐3p on cell migration. (E) The Flow Cytometry assay analysed the effect of LINC02202 on cell apoptosis. *p < 0.05, **p<0.01.

In our study, we proceeded to transfect inhibitors or mimics specific for miR‐526b‐3p into A375 and SKMEL‐5 cells and examined the expression levels of miR‐526b‐3p 72 h post transfection (Figure 4A,B). We observed that miR‐526b‐3p mimics suppressed cell growth, while the miR‐526b‐3p inhibitor group showed promoted growth (Figure 4C). Additionally, the inhibition of miR‐526b‐3p promoted melanoma cell migration, whereas overexpression of miR‐526b‐3p suppressed melanoma cell migration (Figure 4D). Furthermore, the cell apoptosis rate was lower when miR‐526b‐3p was downregulated, but higher when miR‐526b‐3p was induced (Figure 4E). These findings collectively indicate that overexpression of miR‐526b‐3p can suppress melanoma progression.

### 3.5 MiR‐526b‐3p could target XBP1

**FIGURE 5:**
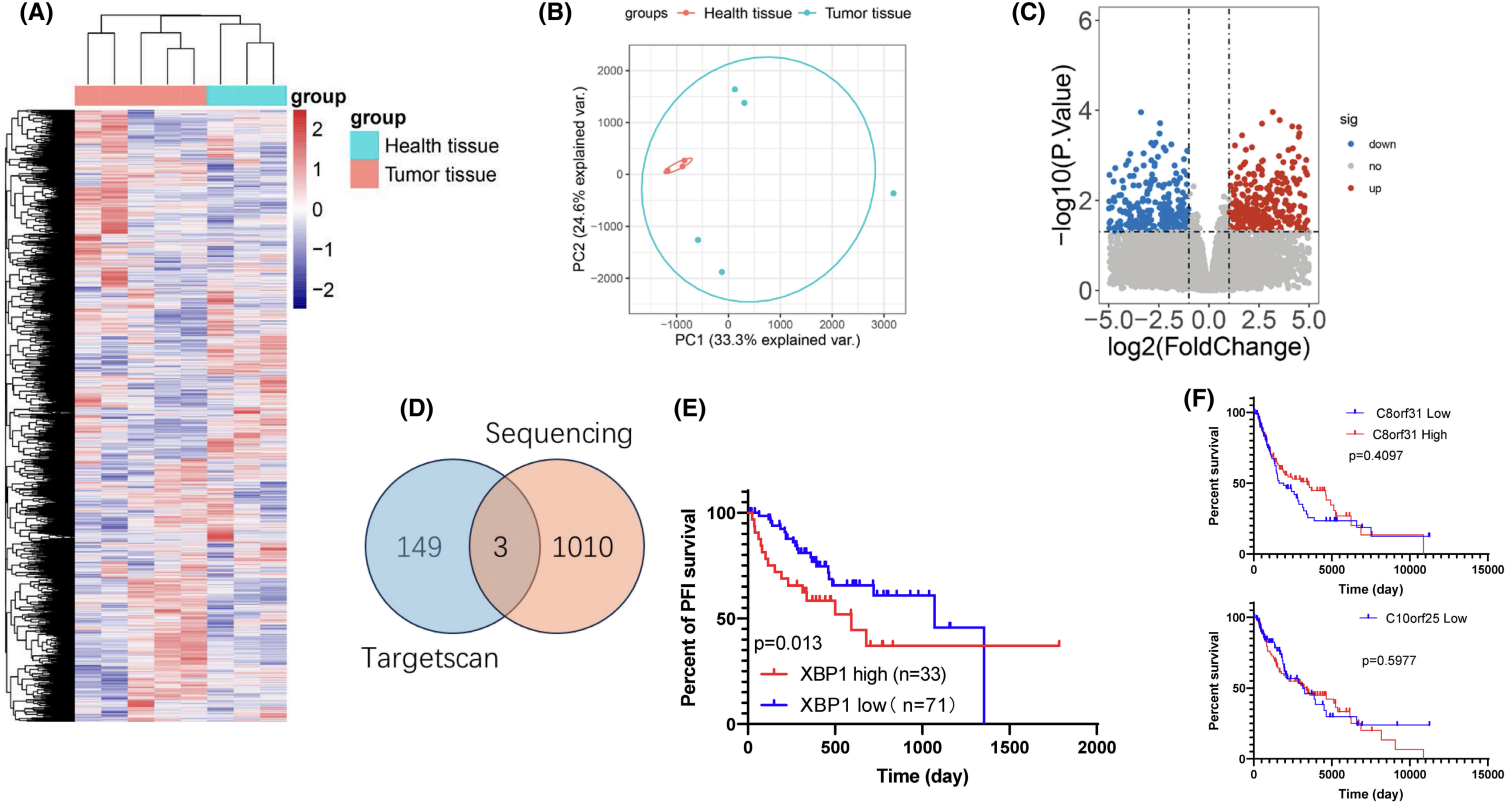
MiR‐526b‐3p could target XBP1. (A) The heatmap shows an overview of differentially expressed genes. (B) PCA analysis the difference of mRNA between the melanoma and melanocytic nevi. (C) Volcano plot analysis differentially expressed mRNAs in melanoma patients and the melanocytic nevi group. (D) Venn analysis of sequence data with targetscan. (E) The survival analysis the correction of XBP1 prognosis by Kaplan–Meier, data obtained from TCGA. (F) The survival analysis the correction of C8orf31 and C10orf25 prognosis by Kaplan–Meier, data obtained from TCGA.

In our study, we conducted an analysis of mRNA expression differences in first‐related genes by analysing our sequencing results. A heatmap was generated to provide an overview of the differentially expressed genes (Figure 5A). PCA analysis was performed to visualize the differences in mRNA expression between melanoma and melanocytic nevi (Figure 5B). Additionally, a volcano plot analysis was conducted to identify differentially expressed mRNAs in melanoma patients compared to the melanocytic nevi group (Figure 5C). By aligning the sequencing data with TARGETSCAN analysis, we identified three genes of interest: XBP1, c8orf31 and c10orf25 (Figure 5D). Survival analysis based on TCGA data demonstrated that XBP1 was positively correlated with poor prognosis (Figure 5E, *p* = 0.013). However, there was no observed correlation between the expression of c8orf31 and c10orf25 and survival. Consequently, we further investigated the regulatory mechanism of XBP1 as a target gene.

### 3.6 LINC02202 promotes melanoma progression by miR‐526b‐3p/XBP1 axis

**FIGURE 6:**
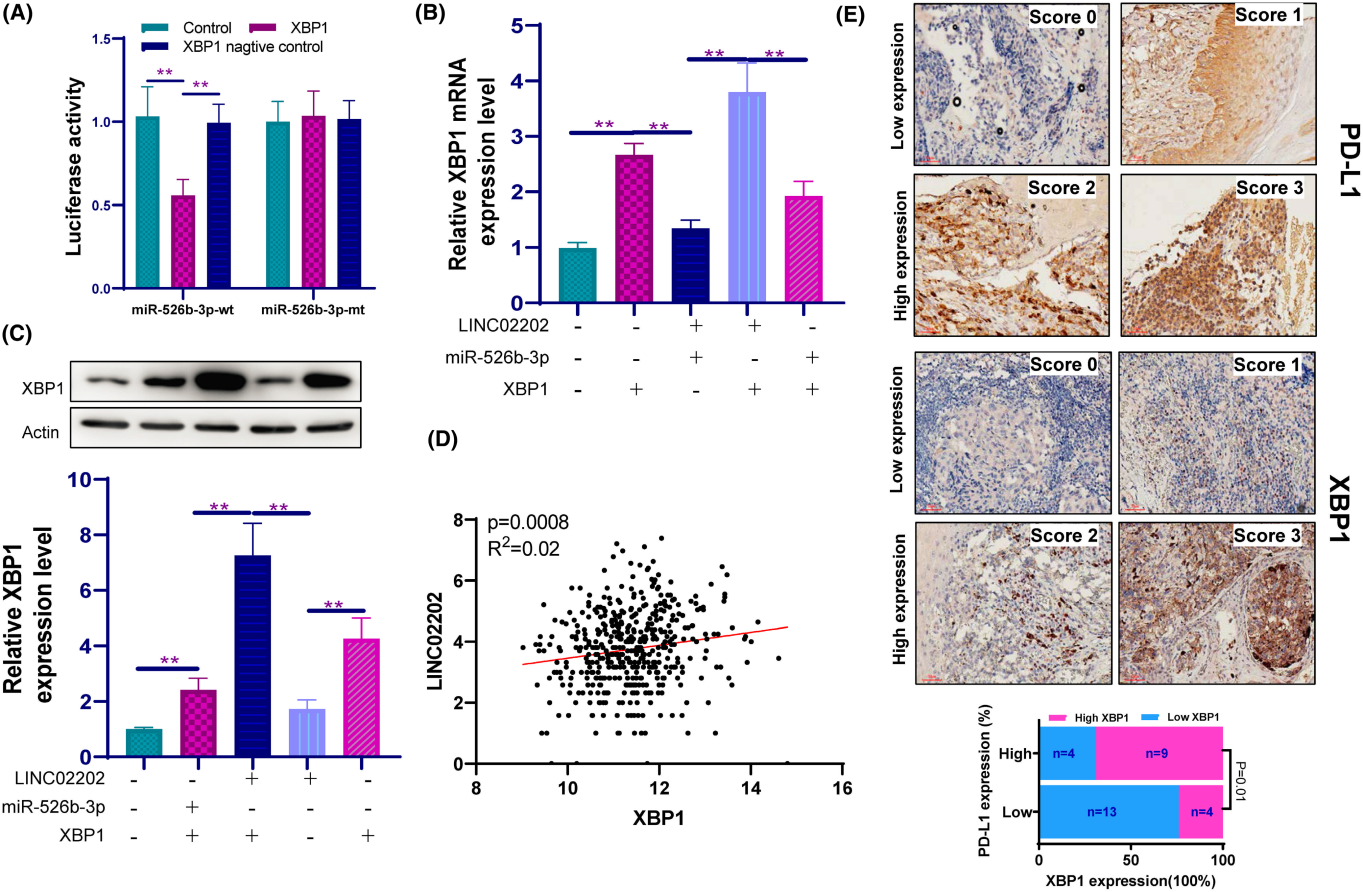
LINC02202 could regulate miR‐526b‐3p/XBP1 axis. (A) The luciferase reporter assay for miR‐526b‐3p that directly targets the 3'UTR of XBP1. (B) The RT‐PCR detected that LINC02202 could regulate miR‐526b‐3p/XBP1 axis. (C) The Western blotting detected that LINC02202 could regulate miR‐526b‐3p/XBP1 axis. (D) Correlation analysis between LINC02202 and XBP1 expression. (E) Immunohistochemical detection of XBP1 and PD‐L1 expression in human melanoma tissue. **p<0.01.

To investigate the regulatory relationship between miR‐526b‐3p and XBP1, we conducted a luciferase reporter assay that confirmed the direct targeting of the 3'UTR of XBP1 by miR‐526b‐3p. This was demonstrated by significantly lower luciferase activity in the miR‐526b‐3p mimics+XBP1‐wt group (Figure 6A). These findings indicate that miR‐526b‐3p inhibits XBP1 expression by directly targeting its 3'UTR.We also examined the relationship between LINC02202 and XBP1. Our results showed that not only miR‐526b‐3p can affect the expression of XBP1, but LINC02202 also influences the expression of XBP1. Moreover, the upregulation of miR‐526b‐3p can block the expression of XBP1 induced by LINC02202 at both the mRNA (Figure 6B) and protein (Figure 6C) levels. Furthermore, we observed a positive correlation between the expression of LINC02202 and XBP1 (Figure 6D). Finally, we tested the correlation between XBP1 and PD‐L1 expression using 30 human samples (patient information in Table 2) and found a positive correlation between XBP1 and PD‐L1 expression (Figure 6E). In summary, LINC02202 can regulate XBP1 expression by targeting miR‐526b‐3p.

**TABLE 2:** General characteristics of malignant melanoma patients.

Variable	Total (*n* = 30)	XBP1 expression		*p*
		High	Low	
Gender				
Female	16	6	10	0.491
Male	14	7	7	
Age				
≥60	18	10	8	0.098
<60	12	3	9	
Stage				
I/II	20	8	12	0.602
III/Iva	10	5	5	
PD‐L1				
High	13	9	4	0.012
Low	17	4	13	

### 3.7 Inhibiting LINC02202/XBP1 can increase the efficacy of PD‐1 immunotherapy in melanoma

**FIGURE 7:**
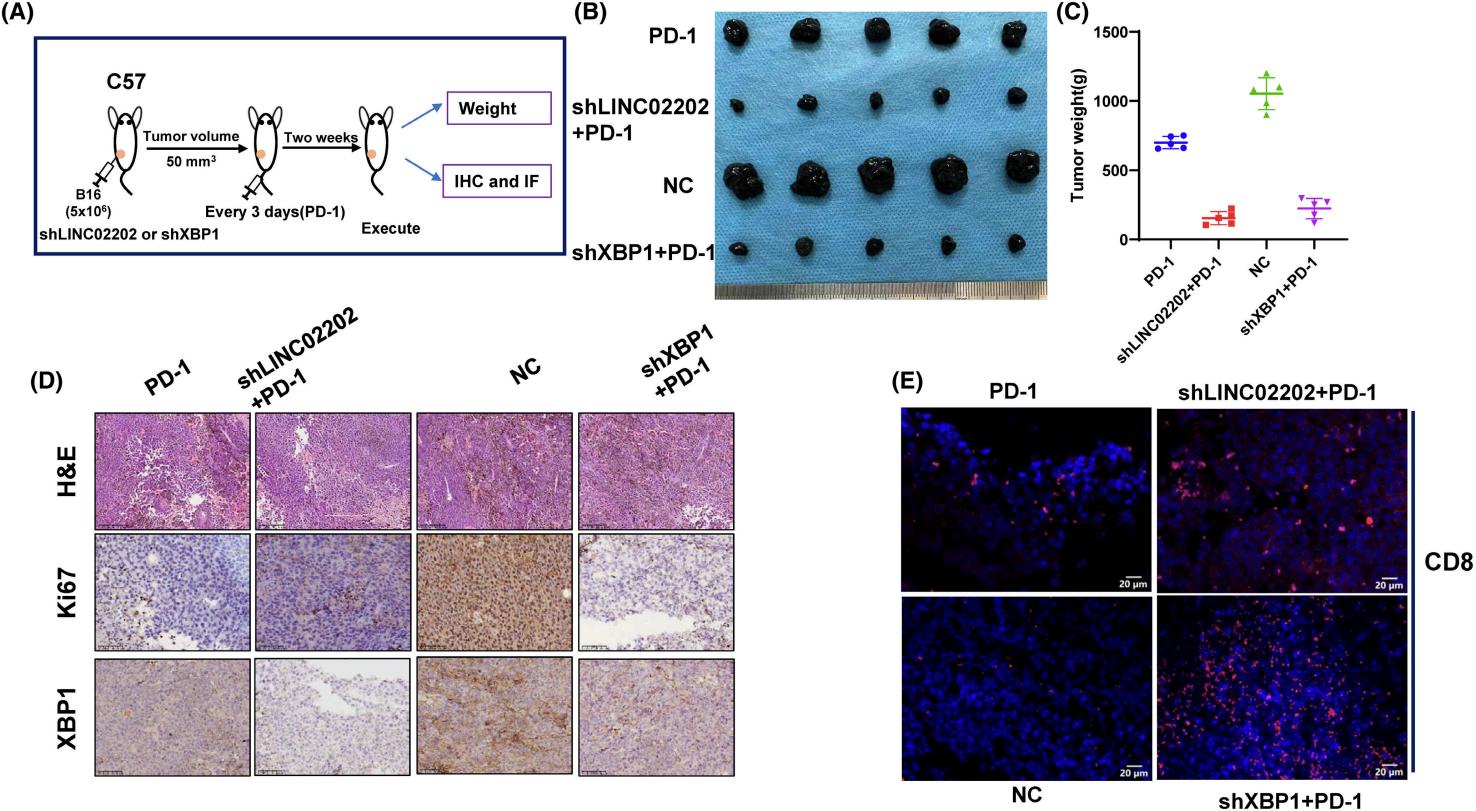
LINC02202 could regulate XBP1 axis affects immune killing in vivo. (A) Animal experimental design diagram. (B) Tumour image. (C) Tumour weight. (D) The IHC detection of ki‐67 and XBP1expression. (E) IF detection of CD8 expression.

In our animal experiments, we observed that tumour volume and tumour weight were significantly decreased when shLINC02202/shXBP1 were combined with anti‐PD‐1 treatment (Figure 7A,B). On the contrary, overexpression of LINC02202 promoted tumour growth. Furthermore, inhibiting XBP1 weakened the expression of Ki‐67, which was induced by LINC02202 (Figure 7C). Additionally, shXBP1 enhanced the infiltrate of CD8+ T cells (Figure 7D). The expression of LINC02202 and XBP1 showed in Figure S1. Overall, our findings suggest that LINC02202 can promote melanoma progression and inhibit T cell immune killing of melanoma cells through the miR‐526b‐3p/XBP1 pathway.

## 4 DISCUSSION

Indeed, long noncoding RNAs (lncRNAs) have been recognized as important regulators of various cellular processes, including those involved in cancer development and progression, such as melanoma. Aberrant expression of lncRNAs has been implicated in the pathogenesis of melanoma, and their dysregulation can impact cellular processes like proliferation, differentiation, migration, invasion and apoptosis.^13^, ^14^, ^15^, ^16^

The tissue‐ and disease‐specific expression patterns of lncRNAs make them potential specific markers for non‐invasive diagnosis of diseases, including cancer.^17^ Although lncRNAs are present at higher levels than mRNAs, their distinct expression profiles and regulatory mechanisms make them promising candidates for diagnostic and therapeutic applications.^18^, ^19^, ^20^ Melanoma, being a well‐studied and genetically defined tumour, has been used as a model to investigate the growth arrest of lesions following oncogene mutations. The immune system and the tumour microenvironment play crucial roles in controlling the growth of moles and preventing their progression to melanoma.^21^, ^22^ Studies have shown that the rate of progression from mole to melanoma may be slightly higher in transplant recipients, suggesting the involvement of the immune system.^23^ Histological evidence indicates that melanomas exhibit stronger lymphocytic infiltration and cytotoxic responses compared to pigmented nevi.^22^, ^23^ However, the interactions between immune cells and moles under steady‐state conditions and their role in inhibiting growth and preventing transformation into melanoma are not fully understood.^21^, ^22^, ^23^, ^24^ Further investigation is required to elucidate these interactions and mechanisms.

Research has found that IRE1 α/KIRA8, an inhibitor of the XBP1 signalling pathway, can significantly inhibit tumour growth and has a better therapeutic effect when combined with PD‐1 antibodies.^25^ Immunotherapy has shown promising results in the treatment of melanoma, but many mechanisms underlying its efficacy are still unclear. In our study, we found that LINC02202 is highly expressed in melanoma and can regulate the expression of miR‐526b‐3p/XBP1/PD‐L1 axis. In vitro experiments revealed that disturbance of LINC02202 inhibited the viability, proliferation and metastasis of melanoma cells (SKMEL‐5 and A375). Animal experiments further confirmed that LINC02202 may regulate the efficacy of immunotherapy in melanoma through the XBP1 pathway.

In summary, our findings suggest that LINC02202 plays a role in activating melanoma cell viability and metastasis while inhibiting apoptosis. It promotes melanoma progression through the miR‐526b‐3p/XBP1/PD‐L1 axis. Inhibition of LINC02202 may represent a potential therapeutic strategy for the treatment of melanoma.

## AUTHOR CONTRIBUTIONS

**Yuanyuan Shang:** Conceptualization (lead); data curation (equal); funding acquisition (lead); investigation (lead); writing – original draft (equal); writing – review and editing (lead). **Haiqian Yang:** Formal analysis (equal); software (equal). **Jian Cui:** Project administration (equal); visualization (equal). **Lipeng Wang:** Formal analysis (equal); investigation (equal); validation (equal). **Le wang:** Investigation (equal); resources (equal); writing – original draft (equal). **Yuan Wang:** Data curation (equal); project administration (equal); visualization (equal). **Miao‐Miao Zhao:** Funding acquisition (equal); methodology (equal). **Pei‐Yao Yu:** Formal analysis (equal); resources (equal); writing – original draft (equal); writing – review and editing (equal). **Hui Qiao:** Writing – original draft (equal); writing – review and editing (lead). **Wen‐Jun Yang:** Funding acquisition (equal); writing – original draft (lead); writing – review and editing (equal).

## FUNDING INFORMATION

This study was sponsored by the the Natural Science Foundation of NingXia (2020AAC03408) and (2023AAC02061); Innovative and Entrepreneurial projects for returnees of NingXia (Ningren letter [2021] No. 5), The First‐Class Discipline Construction Founded Project of Ningxia Medical University.

## CONFLICT OF INTEREST STATEMENT

The authors declare that they have no competing interests.

## Supporting information


Figure S1.

## Data Availability

The datasets used and analysed during the current study are available from the corresponding author on reasonable request.
