# Supplementary material for: Transcriptomics analysis of LINC02202/XBP1 axis in melanoma: Implications for drug targeting and PD‐1 monoclonal antibody efficacy
Source: J Cell Mol Med. 2024 Mar 23;28(8):e18247. doi: 10.1111/jcmm.18247 (PMC10960173; doi:10.1111/jcmm.18247)
Supplement: Supplementary file 1 — Figure S1. [file JCMM-28-e18247-s001.docx]

Supplementary data

We detected the expression LINC02202 and XBP1 in tumor tissue of mouse. We found that the expression of LINC02202 and XBP1 knockdown by shRNA.


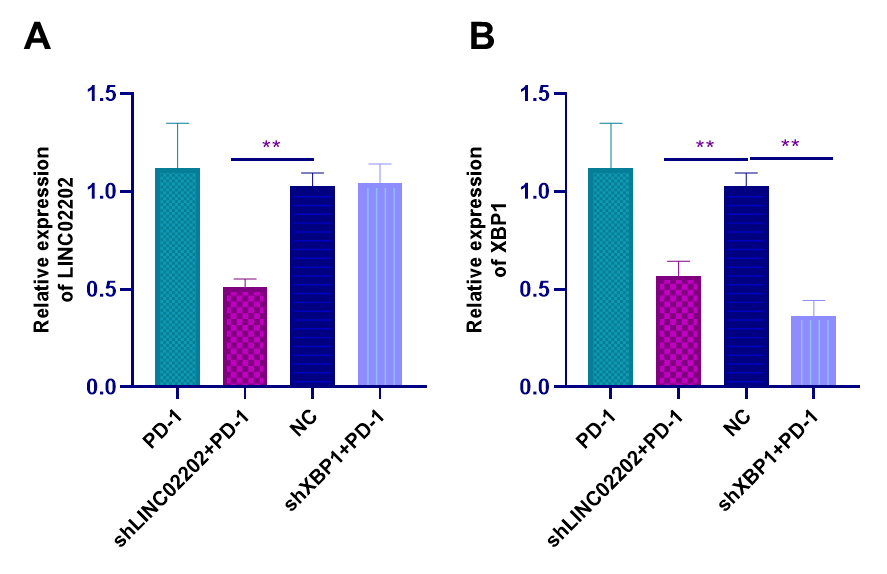


Figure s1. A: RT-PCR detection of LINC02202 expression in mouse tumor tissue; B: RT-PCR detection of XBP1 expression in mouse tumor tissue.
